# Non-leprosy related dapsone hypersensitivity syndrome: A case report and literature review

**DOI:** 10.1097/MD.0000000000043073

**Published:** 2025-07-04

**Authors:** Hang-Qi Zhu, Si-Hua Jiang, Guo-Lin Song

**Affiliations:** a The Second Hospital Affiliated to Guizhou University of Traditional Chinese Medicine, Guiyang, Guizhou, China.

**Keywords:** dapsone hypersensitivity syndrome, hypersensitivity conditions, immunological, leprosy

## Abstract

**Rationale:**

Dapsone is used for treating infectious and immunological disorders, but it may cause dapsone hypersensitivity syndrome (DHS), a serious concern for medical staff. However, in non-leprotic patients, the manifestations of DHS can be atypical and various, leading to diagnostic challenges and potential delays in treatment initiation, which requires attention.

**Patient concerns:**

A 17-year-old Chinese male developed DHS after 3-week dapsone treatment for vasculitis, showing the risk of dapsone use in young patients.

**Diagnosis:**

DHS symptoms include fever, rash, and lymphadenopathy, which can be life-threatening. Diagnosis for this patient was based on symptom recognition, medical history review, physical examination, and the link between dapsone intake and symptom onset.

**Interventions:**

Once diagnosed, dapsone was withdrawn immediately. Corticosteroids were given to reduce inflammation, and antipyretics and anti-histamines were used for symptom relief.

**Outcomes:**

After treatment, the patient improved. Fever subsided quickly, the rash resolved in a week, and lymphadenopathy shrank. Follow-up showed full recovery with no symptom recurrence.

**Lessons:**

This report details a case and reviews published cases of DHS. Summarizing their features aims to improve diagnostic accuracy and management strategies, thus helping healthcare providers handle similar cases better.

## 1. Introduction

Dapsone (4,4´-diaminodiphenyl sulfone) has been a mainstay in leprosy treatment for over 70 years. Despite its generally recognized safety profile, it is associated with potential side effects.^[1]^ One of the most serious and life-threatening complications is dapsone hypersensitivity syndrome (DHS). DHS is characterized by fever, rash, lymphadenopathy, eosinophilia, jaundice, splenomegaly, and pedal edema, often accompanied by hepatitis, hemolytic anemia, and atypical lymphocytosis, even at low doses.^[2]^

In addition to being used for leprosy, dapsone is now widely employed in the treatment of various infectious, immune, and hypersensitivity disorders.^[3]^ In patients without leprosy, DHS may present with milder symptoms and a better prognosis, yet this variability complicates diagnosis, potentially leading to misinterpretation.^[4]^

We present a case of a 17-year-old Chinese man who developed DHS after 3 weeks of dapsone therapy for rash. We provide an overview of the presentations, diagnosis, and management of DHS in the non-leprosy patients.

## 2. Case report

A 17-year-old Chinese male was referred for evaluation of a suspected vasculitis-related rash. He presented with a 10-day history of high fever, malaise, productive cough, and pruritic rash. Initially diagnosed with dermatitis, he had been prescribed oral dapsone 100 mg daily by a dermatologist. Three weeks later, he consulted his primary care physician with complaints of high fever, malaise, lymphadenopathy, maculopapular skin rash, and painful oral mucosal lesions. Subsequently, he was referred to our infectious disease clinic with a suspected diagnosis of adult infectious mononucleosis.

**Figure 1.**
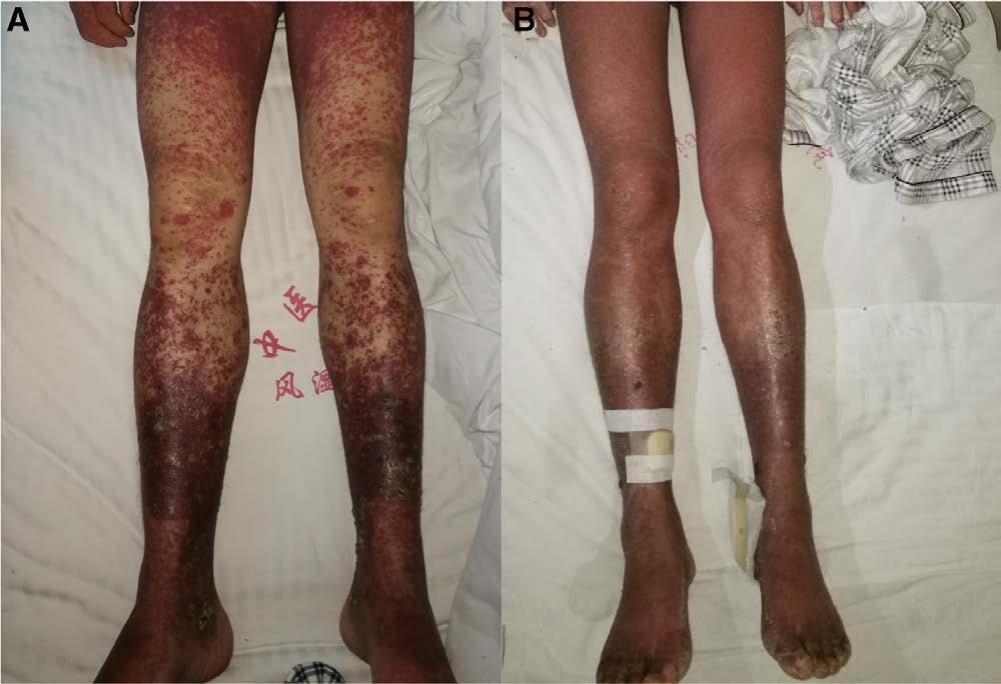
(A) Before treatment: A large number of rashes can be seen in the limbs and trunk. (B) 3 weeks after treatment: the rash began to disappear.

On admission, the patient presented with a temperature of 39.6 °C, blood pressure of 100/60 mm Hg, and a heart rate of 112 beats/min. He displayed dark red circular or oval-shaped rashes on his forehead, neck, trunk, back, and limbs, some of which were partially confluent. The skin appeared slightly warm with uneven surfaces, non-blanching on palpation, accompanied by tenderness and mild itching. The ankles were most severely affected, with visible ulcers (Fig. 1). Pharyngeal examination revealed hyperemia. Multiple tender bilateral cervical lymph nodes measuring 1 to 2 cm were palpable. Lung and heart sounds were unremarkable, except for slight wheezing. The slightly softened and enlarged liver and spleen can be palpated 2 cm below the rib margin. There was no evidence of ascites. Initial management included discontinuation of dapsone and application of physical cooling. Blood samples were collected for routine tests, including serological testing for mononucleosis.

A complete blood count revealed the following: hemoglobin 15.1 g, hematocrit 32.6%, white blood cell counts 29,860/mm^3^ (comprising neutrophils 48.3%, lymphocytes 27.3%, and monocytes 24.4%), platelet count 217,000/mm^3^, and erythrocyte sedimentation rate of 5 mm in the first hour. The liver function tests were abnormal: direct bilirubin 1.46 mg/dL, indirect bilirubin 0.90 mg/dL, aspartate aminotransferase 106 U/L, alanine aminotransferase 263 U/L, alkaline phosphatase 159 U/L, serum albumin 3.38 g/dL, and prothrombin time 17.1 seconds. Viral hepatitis serology (IgM antibody to hepatitis A antigen, hepatitis B surface antigen, and hepatitis C antibody) were negative. Despite evident hematuria, urea, creatinine, uric acid, and electrolytes in blood were within normal limits.

The serological test for rubella virus revealed elevated levels of specific IgG and negative results for specific IgM. A mononucleosis spot test was also negative. Blood and sputum cultures were both negative for bacterial growth. The culture from the wound secretion at the ankle showed growth for *Staphylococcus aureus*. Levels of complement components C3 and C4 were low (C3, 17.2 mg/dL and C4, 3.9 mg/dL) and autoimmune screens for antinuclear antibodies, anti-double-stranded DNA antibodies, anti-smooth muscle antibodies, and anti-liver-kidney-microsome antibodies were negative. Abdominal ultrasound showed uniform liver enlargement with a slight increase in echo texture, and there was no evidence of portal hypertension or biliary obstruction. His chest radiograph showed no significant abnormalities.

The clinical presentation and laboratory findings of the patient were indicative of DHS accompanied by leukemoid reaction. He was placed on a regular diet and commenced glucocorticoid therapy with oral prednisolone at a dosage of 40 mg per day. His clinical condition improved rapidly, and the rash is also decreasing. Serial laboratory monitoring demonstrated progressive normalization of all abnormal parameters over the treatment period (Table 1). Within 3 weeks, his laboratory parameters returned to normal levels. We gradually tapered the corticosteroid treatment over an 8-week period, after which he was discharged. Subsequent follow-up examinations and laboratory results remained within normal limits.

**Table 1:** Longitudinal changes in laboratory test results (June 19–July 17, 2023).

Test parameter	Reference range	June19	June20	June23	June25	June26	June28	July 1	July 6	July10	July17
WBC (×10^9^/L)	3.5–9.5	**29.86**	**33.55**	**29.73**	**–**	**18.19**	**14.01**	**12.54**	**11.33**	**10.46**	**10.11**
NEUT% (%)	40–75	38.3	37.1	28.9	**–**	30.9	**44.3**	**58.0**	**46.8**	**49.6**	**42.8**
NEUT# (×10^9^/L)	1.8–6.3	**11.46**	**12.45**	**8.62**	**–**	5.62	6.21	**7.26**	5.3	5.18	4.33
MONO% (%)	3–10	**24.4**	**25.2**	**19.4**	**–**	**12.9**	9.4	8.5	**12.4**	**10.2**	**12.3**
MONO# (×10^9^/L)	0.1–0.6	**7.28**	**8.44**	**5.76**	**–**	**2.35**	**1.32**	**1.07**	**1.41**	**1.07**	**1.25**
ESR (mm/h)	0–15	5	**–**	**–**	**–**	**–**	**–**	5	**–**	**–**	**–**
AST (U/L)	0–42	**106**	**–**	**158**	**284**	**–**	**102**	40	30	24	21
ALT (U/L)	0–42	**263**	**–**	**325**	**534**	**–**	**415**	**211**	**70**	**58**	24
IgG (g/L)	6.5–16	6.44	**–**	**–**	**–**	**–**	**–**	**–**	**–**	**–**	**–**
IgM (g/L)	0.5–3	0.49	**–**	**–**	**–**	**–**	**–**	**–**	**–**	**–**	**–**
PCT (ng/mL)	<0.05	**–**	**2**	**–**	**–**	**–**	**–**	**0.09**	**–**	**–**	**–**
CK (U/L)	0–255	45	**–**	**–**	165	**–**	**547**	**–**	**–**	**–**	**–**
D-DIM (mg/L)	0–0.55	**–**	**–**	**–**	**6.3**	**–**	**4.01**	**1.24**	**–**	**–**	**0.59**

Abnormal values are highlighted in bold.

ALT = alanine aminotransferase, AST = aspartate aminotransferase, CK = creatine kinase, D-DIM = D-dimer, ESR = erythrocyte sedimentation rate, IgG = immunoglobulin G, IgM = immunoglobulin M, MONO% = monocyte percentage, MONO# = absolute monocyte count, NEUT% = neutrophil percentage, NEUT# = absolute neutrophil count, PCT = procalcitonin, WBC = white blood cell count, **–** = not tested.

## 3. Discussion

Dapsone, widely used in medicine for its anti-inflammatory and immunomodulatory effects, can potentially induce drug-induced hypersensitivity syndrome/drug reaction with eosinophilia and systemic symptoms.^[5]^ Lowe and Smith first documented adverse reactions associated with dapsone in 1949, noting that approximately 12% of patients developed a rash after initiating treatment.^[4]^ In 1981, Tomecki and Catalano introduced the term DHS to describe this syndrome, characterized by fever, rash, lymphadenopathy, eosinophilia, jaundice, splenomegaly, and pedal edema.^[6]^ Other potential manifestations include hemolysis, neutropenia, methemoglobinemia, lymphocytosis, and toxic epidermal necrolysis. DHS typically manifests later than other drug eruptions (approximately 2–8 weeks after initiation of treatment). The diagnostic criteria for DHS proposed by Richardus and Smith in 1989 remain in use today, requiring the major symptoms appearing within 8 weeks of starting treatment, which resolve upon discontinuation of the drug.^[7]^

DHS primarily occurs in Asia^[5]^ and affects both children and adults,^[8]^ with an average age of 35.2 years (range 5–83 years) and a male-to-female ratio of 1.5:1.^[9]^ In China, the incidence of DHS among dapsone-treated patients is 1.5%, with a mortality rate of 9.6%.^[10]^ Among leprosy patients, approximately 2% develop DHS, with a mortality rate of 12.5%. Non-leprosy patients generally have a better prognosis than leprosy patients.^[11]^ To further understand the specific characteristics of non-leprosy-related DHS, we systematically reviewed all relevant cases indexed on PubMed using the search term “Dapsone Hypersensitivity Syndrome” (Table 2).^[1,8,9,12–18]^ DHS tends to occur earlier in non-leprosy patients, often within the first 3 weeks, and their symptoms are relatively mild, with some cases presenting primarily with fever, which can lead to under-recognition. Glucocorticoids demonstrate significant efficacy in the treatment of non-leprosy-related DHS.Similarly, in our report, the patient developed a range of symptoms predominantly characterized by fever and lymphadenopathy, along with mild hepatosplenomegaly. Corticosteroid treatment was effective.

**Table 2:** Characteristics of 11 patients with DHS.

Source	Country	Age (y)	Sex	Rash delay (weeks)	Symptom (symptoms associated with rash)	Treatments	Response to therapy
Sener O^[9]^	Turkey	21	F	4	Fever, lymphadenopathy	Steroids, HZ	CR
Vinod KV^[12]^	India	17	M	3	Fever, lymphadenopathy, hepatitis	Steroids	CR
Kim GW^[13]^	Korea	8	F	3	Fever, hematuria, lymphadenopathy	Steroids	CR
Kosseifi SG^[1]^	USA	21	F	1	Fever, jaundice	Steroids	CR
Rim MY^[14]^	Korea	36	F	2	Fever	Steroids	CR
Leslie KS^[15]^	UK	55	F	1	Fever	Steroids	CR
Zhu KJ^[16]^	China	45	F	5	Fever, dyspnea, syncope	Steroids, IvIg	CR
Schulkes KJG^[17]^	Netherlands	55	F	4	Fever, dyspnea	Steroids	CR
Teo RY^[18]^	Malay	22	M	12	Fever, abdominal pain	Steroids	CR
Zurina Z^[8]^	Malaysia	12	F	7	Fever, diarrhea	Steroids	CR
Our patient	China	17	M	3	Fever, lymphadenoma	Steroids	CR

CR = complete response, DHS = dapsone hypersensitivity syndrome, HZ = hydroxyzine, IvIg = intravenous immunoglobulin.

The pathogenesis of DHS is believed to hinge on the metabolic pathways of dapsone. Dapsone is known to undergo acetylation and hydroxylation pathways, with dapsone hydroxylamine believed to be the cause of adverse effects.^[19]^ Knowles et al suggest that this is a combination of Type I, IV, and possibly Type III Gel and Coombs hypersensitivity reactions, as well as selective modification of graft versus host disease mediated by activated T lymphocytes.^[20]^ Recent genome-wide association studies have highlighted a close association between the human leukocyte antigen (HLA)-B13:01 polymorphism and DHS risk in Indian leprosy patients treated with dapsone.^[21]^ HLA genes, known for their high polymorphism in the human genome, play a crucial role in immune responses. HLA-B*13:01 specifically binds dapsone and activates dapsone-specific cytotoxic T lymphocytes in an HLA-B*13:01-dependent manner.^[22]^ It is noteworthy that HLA genes are also closely associated with susceptibility to leprosy,^[23]^ potentially elucidating why DHS is prevalent among leprosy patients and tends to manifest more severely in this group.

Considering the challenges in identifying DHS early, HLA-B*13:01 may play a crucial role in its prevention. Research indicates that the HLA-B*13:01 allele sensitively and specifically predicts DHS (85.5% and 85.7%, respectively), and its absence correlates with a sevenfold decrease in risk.^[10]^ Moreover, this gene is prevalent among Asians, particularly Chinese populations.^[24]^ Screening HLA-B*13:01 positive individuals could significantly reduce the incidence of DHS in Chinese populations, ensuring patient safety.

Generally, DHS is a self-limiting drug reaction. Timely discontinuation of dapsone and cross-reactive medications is crucial for subsequent recovery. In addition, systemic corticosteroids are considered fundamental for treatment.^[5]^ Prednisone or an equivalent dose of 1.0 mg/kg/day is typically initiated as the initial dose for systemic steroid therapy,^[25]^ with rash and fever often beginning to diminish within days thereafter. Adjunct therapies like cyclosporine and immunoglobulin may also confer additional benefits during treatment.^[26,27]^ Due to the long elimination half-life of dapsone (24–30 hours) and its prolonged presence in organs for up to 35 days,^[28]^ it is recommended to undergo at least 1 month of corticosteroid therapy and closely monitor organ function.^[20]^ Even after clinical remission, steroids are necessary for 6 to 8 weeks to prevent recurrence and the development of immune reconstitution inflammatory syndrome.

## 4. Conclusion

This case illustrates the typical manifestations of DHS and summarizes its pathogenesis, treatment, and prognosis, focusing particularly on non-leprosy related DHS. A high level of clinical awareness forms the foundation for early diagnosis, as immediate cessation of medication and early initiation of systemic corticosteroids can lead to rapid recovery and mitigate the risk of severe morbidity or even mortality. HLA-B*13:01 serves as a valuable genetic marker for DHS, holding significant clinical utility in screening and potential therapeutic interventions.

## Author contributions

**Conceptualization**: Si-Hua Jiang.

**Data curation:** Si-Hua Jiang, Guo-Lin Song.

**Formal analysis:** Guo-Lin Song.

**Funding acquisition:** Si-Hua Jiang, Guo-Lin Song.

**Investigation:** Si-Hua Jiang.

**Methodology:** Guo-Lin Song.

**Project administration:** Hang-Qi Zhu, Guo-Lin Song.

**Resources:** Hang-Qi Zhu.

**Software:** Hang-Qi Zhu.

**Supervision:** Hang-Qi Zhu.
